# Malaria and Severe Anemia: Thinking beyond *Plasmodium falciparum*

**DOI:** 10.1371/journal.pmed.1001576

**Published:** 2013-12-17

**Authors:** Roly D. Gosling, Michelle S. Hsiang

**Affiliations:** 1 Malaria Elimination Initiative, Global Health Group, University of California, San Francisco, San Francisco, California, United States of America; 2 Department of Pediatrics, UCSF Benioff Children's Hospital, University of California, San Francisco, San Francisco, California, United States of America

## Abstract

In a linked Perspective, Roly Gosling and Michelle Hsiang discuss the importance of non-falciparum malaria species to regional and global health.

*Please see later in the article for the Editors' Summary*

**Linked Research Article**

This Perspective discusses the following new study published in *PLOS Medicine*:

Douglas NM, Lampah DA, Kenangalem E, Simpson JA, Poespoprodjo JR, et al. (2013) Major Burden of Severe Anemia from Non-Falciparum Malaria Species in Southern Papua: A Hospital-Based Surveillance Study. PLoS Med 10(13): e1001575. doi:10.1371/journal.pmed.1001575

Ric Price and colleagues use hospital-based surveillance data to estimate the risk of severe anemia and mortality associated with endemic *Plasmodium* species in southern Papua, Indonesia.

In this week's *PLOS Medicine*, Ric Price and colleagues compare the burden of anemia in different plasmodia species in a robust hospital-based surveillance study in Eastern Indonesia [1]. The risks of severe anemia associated with non-falciparum and mixed species infections are characterized and compared to no malaria infection and monoinfection with *Plasmodium falciparum*. While the study is observational, and limited in its ability to control for co-morbidities (e.g., geohelminth, bacterial infection), the numbers are impressive, with more than 200,000 outpatient and inpatient episodes with hematological assessment. Their findings reveal a significant burden of severe anemia (defined as hemoglobin less than 5 g/dl) due to *P. vivax*, *P. malariae*, or mixed species infections (adjusted population attributable fraction 12.2%), in addition to *P. falciparum* monoinfection (15.1%). Notably, severe anemia in infants attributable to *P. vivax* was 30.4% compared to 20.5% for *P. falciparum*. Patients with severe anemia were substantially more likely to be admitted to the hospital (adjusted odds ratio, 6.34 [95% CI 6.00–6.69]) and to die (adjusted odds ratio, 5.80 [95% CI 5.17–6.50]).

## Regional and Global Health Significance

*P. falciparum* receives the most attention because it causes the most deaths, largely in Africa, despite *P. vivax* being the most geographically widespread species of human malaria. More than 2 billion people are at risk of contracting *P. vivax*, stretching from Latin America, northern Africa, Arabia, Central Europe, to Asia and the Pacific [2]. It is substantially harder to control than *P. falciparum*, because a wider variety of *Anopheles* species with varied habits spread the infection; and because the dormant liver stage, the *hypnozoite*, can cause relapses, from days to years later and is notoriously hard to treat. There is a misconception that *P. vivax* is a benign disease despite it being well established that *P. vivax* causes severe chronic illness and newer studies linking it to severe illness and death [3]. Mixed-species infections have been under-recognized and the clinical significance not well understood. Studies have shown that when more sensitive molecular detection methods are used, prevalence of mixed infections is upwards of 30% [4]. While prior studies showed concurrent infections of different species to be mutually suppressive, more recent studies, including the study by Price and colleagues, suggest otherwise [1],[5],[6].

Numerous countries have successfully eliminated *P. falciparum* yet continue to battle to eliminate *P. vivax* [7]. The disability-adjusted life years (DALYs) lost due to infection by *P. falciparum* have been a strong argument for global investment in control and elimination of *P. falciparum*, including drug-resistant *P. falciparum* [8]. With the findings from this study, similar economic arguments for *P. vivax* control and elimination can start to be built, supporting an investment case for the elimination of *P. vivax*. Indonesia, where this study took place, is working with 14 other countries in the Asia Pacific, the Asia Pacific Malaria Elimination Network (APMEN) [9], to become malaria free. APMEN pays particular attention to *P. vivax*, which is the dominant species of *Plasmodia* in the region, and is in need of such economic arguments.

## Clinical Management Issues

For severe anemia due to non-falciparum infections, some clinical management issues arise. Patients are likely to require blood transfusion, which carries risk for a variety of transfusion-related reactions and transfusion-associated infections such as HIV and hepatitis [5]. Furthermore, blood transfusions are not readily available in resource-poor settings. As for iron supplementation, there has traditionally been reluctance to use iron supplementation in the setting of infection because many pathogens utilize iron for survival and pathogenesis, and removal of free circulating iron seems to be an important part of the human response to infection. However, a recent Cochrane Review [10] examined four studies of iron versus control in the treatment of proven malaria in *P. falciparum* endemic areas and found that treatment of anemia during an acute attack of malaria improves hemoglobin recovery and does not increase the risk of treatment failure or death [10]. More data from high transmission settings with non-falciparum malaria are needed [5].

For *P. vivax* specifically, antimalarial treatment and anemia are inextricably connected. Radical cure requires the use of a hypnozoitocidal agent, of which the only one widely available is primaquine. This 8-aminoquinoline causes hemolysis that can be severe in patients with underlying glucose-6-phosphate dehydrogenase (G6PD) deficiency, found commonly in malaria endemic areas [11]. The severity of the hemolysis is dependent on the severity of the deficiency. Unfortunately, convenient and reliable quantitative or qualitative tests for G6PD deficiency are not available. The fear of using primaquine in an already anemic patient is likely to lead to its underuse and contributes to the relapses and the resultant chronic anemia from *P. vivax*.

## Implications for Prevention Measures

On a population level, chronic anemia is well known to cause poor growth and cognitive deficits in children. Chronic anemia can also predispose to severe anemia in the setting of a malaria infection. Community preventative treatment of children with iron supplementation to prevent anemia in *P. falciparum* endemic areas was cautioned following a study of routine iron and folate supplementation to children in Zanzibar, Tanzania that showed an association with severe illness and death related to iron supplementation and *P. falciparum* infection [12],[13]. However, the aforementioned Cochrane Review examined 13 trials from predominantly falciparum endemic areas and found no significant difference in clinical malaria or deaths for individuals treated with iron alone versus placebo, though in trials in which malaria surveillance and treatment was not provided, the risk for clinical malaria was higher in individuals treated with iron or iron plus folic acid [10]. More data from high transmission settings with non-falciparum malaria are needed. In a similar setting to Timika, Indonesia, a study in Papua New Guinea of intermittent preventative treatment of infants (IPTi) with antimalarials given to infants and children at the time of routine immunizations showed a decrease in the risk of severe anemia by 51%–87%, echoing findings in two previous African trials [14],[15]. Increasing the age range of IPT to include older children, especially in seasonal settings, may further improve outcomes [16].

There is also a need to evaluate the impact of standard and new malaria control interventions on morbidity and mortality associated with non-falciparum malaria. Community benefits of vector control using insecticide-treated bed nets (ITNs) for *P. vivax* are less well established in the region, where outdoor biting and resting *Anopheles* are common. More research is needed on methods to reduce transmission of all plasmodia species in the Asia Pacific, such as the use of active case detection to find asymptomatic infections, drug based strategies including IPT regimens, mass drug administration to reduce the reservoir of human infections, development of sensitive rapid diagnostic tests for *P. vivax* or other non-falciparum species, and alternative vector control methods [7].

The study by Price and colleagues requires our thinking to expand beyond *P. falciparum*, to previously under-recognized, but important, clinical and public health issues related to the other human infecting plasmodia. Let us hope that investment follows suit.
